# A decade of research on genetic privacy: the findings of the GetPreCiSe Center at Vanderbilt University

**DOI:** 10.3389/fgene.2025.1629386

**Published:** 2025-08-21

**Authors:** Christopher Slobogin, Karli Tellis, Ellen Wright Clayton, Jay Clayton, Ayden Eilmus, Bradley A. Malin

**Affiliations:** ^1^ Vanderbilt Law School, Nashville, TN, United States; ^2^ Center for Biomedical Ethics and Society, Vanderbilt University Medical Center, Nashville, TN, United States; ^3^ Department of Pediatrics, Vanderbilt University Medical Center, Nashville, TN, United States; ^4^ Department of Health Policy, Vanderbilt University Medical Center, Nashville, TN, United States; ^5^ Department of English, Vanderbilt University, Nashville, TN, United States; ^6^ Division of Medical Ethics, NYU Grossman School of Medicine, New York, NY, United States; ^7^ Department of Biomedical Informatics, Vanderbilt University Medical Center, Nashville, TN, United States; ^8^ Department of Computer Science, Vanderbilt University, Nashville, TN, United States

**Keywords:** genetic privacy, transdisciplinary, law, ethics, sociotechnical, media

## Abstract

Research carried out by Vanderbilt University’s and Medical Center’s federally-funded transdisciplinary, highly interactive GetPreCiSe Center in Excellence for ELSI research on genomic privacy—involving over 40 scholars across computer and social sciences, law, and the humanities—is summarized by dividing the work into five categories: (1) the nature of risks posed by collection of genetic data; (2) legal and scientific methods of minimizing those risks; (3) methods of safely increasing the scope of genetic databases; (4) public perceptions of genetic privacy; and (5) cultural depictions of genetic privacy. While this research shows that the risk of unauthorized re-identification is often over-stated, it also identifies possible ways privacy can be compromised. Several technical and legal methods for reducing privacy risks are described, most of which focus not on collection of the data, but rather on regulating data security, access, and use once it is collected. Researchers also collected empirical data assessing the public’s views on genomic privacy. This research indicated that public concern about genetic privacy may be no greater than concern about financial and other types of privacy and often varies depending on the context in which the information is accessed. More generally, the research suggested that privacy experts may underestimate the extent to which the public values utility over privacy risk. Finally, both survey data and research on media depictions about genetic science found that worry about genetic research and its impact on privacy and other values appear to grow as the time horizon lengthens and varies significantly based on demography; in particular, minority groups defined by ethnicity and sexual identity are more anxious about genetic disclosures than other groups. Research looking at fan fiction, blog posts, and online book reviews also found that these depictions can have a direct impact on public attitudes about genetic science.

## 1 Introduction

Efforts to improve diagnosis and treatment by taking advantage of advances in genetic science have incentivized the collection of vast amounts of genomic data, which is then analyzed and often shared with doctors, researchers, and various private and government entities. Direct to consumer genetic testing (DTC-GT) companies, hoping to monetize recreational genealogy investigations, have also created large genomic datasets. The existence of this personal information about millions of people in the hands of third parties has raised new and challenging privacy concerns, both legal and technical in nature.

This paper describes the principal findings of Vanderbilt’s Center for Genetic Privacy and Identity in Community Settings (GetPreCiSe) since its founding in 2016. The Center was funded as a Center of Excellence in ELSI Research, a mechanism created by the National Human Genome Research Institute to foster in depth, multi-faceted, and agile research to address emerging ethical, legal, and social issues posed by advances in genomics and the changing social environment. When GetPreCiSe was formed, it was based on the expectation that our transdisciplinary team would 1) define genomic privacy and identity issues throughout society, 2) innovate new ways of framing and investigating these problems, and 3) create techniques to assist policymakers in making evidence-based assessment to enable the responsible collection, use, and sharing of genomic data drawn from communities of individuals. Once we established this framework, the Center was able to rapidly form teams that investigated newly developing problems, such as: 1) the rise of direct-to-consumer genetic testing companies that encourage increased sharing of genetic data in ways consumers do not completely grasp, including use of their data by law enforcement agencies interested in tracking down violent crime offenders, such as the Golden State Killer, 2) the novel privacy threats that Supreme Court decisions (e.g., *Dobbs v. Jackson Women’s Health Organization*) posed to genomic data collected and shared in connection with reproductive health, 3) the emerging artificial intelligence techniques that have the potential to exacerbate genomic data re-identification risks, and 4) the increasing representation of genetic privacy in TV, film, and popular culture. While such problems could have been investigated through traditional grant-sponsored research, the process of formulating, applying, and waiting for funding decisions would mean that such projects would have been stalled for over a year after the occurrence of the initiating events. By contrast, our CEER was able to organize a team and begin a new investigation much more rapidly, often completing our studies before a traditional grant would even have been considered for funding.

The research reported here on these and related topics was generated by a diverse group of over 40 scholars, involving faculty and graduate and undergraduate students from a wide range of disciplines, including anthropology, computer and data science, economics, history, law, literature, medicine, and sociology, who engaged with each other throughout the lifetime of the project and whose work evolved in response to events. While the GetPreCiSe group was transdisciplinary, researchers used their own discipline-specific methods and published results in their own disciplinary journals. The goal was not to integrate methods across disciplines or to harmonize data from different sources. Rather each researcher approached different facets of the problem from different angles. For example, data scientists explored the strengths and limitations of various technical strategies to protect privacy in data collections; social scientists employed surveys, focus groups, discourse analysis, and qualitative or quantitative analysis to understand public opinion about various aspects of genetic privacy; legal scholars analyzed legislation and court cases; bioethicists and policy experts explored the social and political dimensions raised by genetic privacy; and humanists sought to understand how culture shaped public attitudes toward genetic privacy by examining the depiction of genetics in literature, films, TV, fandom, and social media. Through frequent meetings of the Center, researchers received input from others and shared findings, a process that often inspired new questions and new collaborations. But seeking consensus was an endpoint of these investigations, not a methodological starting point.

The focus of the Center was on genetic privacy in the social, economic, political, and cultural context of the United States. Early on, it was determined that an international comparative perspective would produce too many variables for the Center to encompass. Not only do social and cultural expectations of privacy and legal frameworks and policy environments vary across nations in North and South America, Europe, Asia, Africa, and the Middle East, they often do so in ways that differ dramatically from those in the United States. This unwieldiness was dramatized in 2016, the year the Center was founded, when the European Union passed the General Data Protection Regulation, radically altering the playing field for genetic privacy in the EU but not in countries in other regions, thereby complicating international collaboration. Hence, while our work was often informed by that of investigators aboard, the research discussed below centers primarily on genetic privacy issues in a US context.

Within this domestic focus, the Center’s research attempted to 1) evaluate the nature and impact of threats to privacy and identity in genomic data, both real and perceived; 2) measure the efficacy of efforts—by communities, Institutional Review Boards, research and other data collection entities, and the law—to protect privacy and identity; 3) develop models that quantify the risk of genetic reidentification and the incentives and disincentives to engage in re-identification; and 4) delineate the types of interventions that can enhance institutional trust and deter privacy intrusions and misuse of genetic data.

Several themes run through this research. One focused on the risk of re-identifying genetic information that has been de-identified by researchers in an effort to protect privacy. While the general conclusion of most of the papers our center produced is that the risk of unauthorized re-identification is often overstated, they all recognize that the threat is real, and many of them outline technical methods of reducing that risk. Our research also provides suggestions about how the law should formally restrict access to this information by employers and other actors, including the government. Most of these restrictions focus not on collection of data, which many believe should be considered a social good and even expanded, but rather on regulating data security, access, and use once it is collected.

Several themes also run through the numerous GetPreCiSe papers that investigate public perceptions about genetic privacy, as measured through surveys, focus groups (which included diverse participants), social media, and popular entertainment. First, the public does not appear to endorse a robust “genetic exceptionalism,” (Garrison et al., 2019), the notion that genetic information raises particularly challenging issues and so requires special treatment; rather, concern about genetic privacy may be no greater than concern about financial and other types of privacy and often varies depending on the context in which the information is accessed. Second, an over-arching finding of the GetPreCiSe scholars who examined media depictions about genetic science found that worry about genetic research and its impact on privacy and other values appears to grow as the time horizon lengthens; our culture seems very concerned about the potential negative impact of genomic manipulations in the distant future, but reacts relatively positively to current efforts to harness that science for health or criminal investigation purposes. Third, trust in the ability to protect genetic privacy varies significantly based on demography; in particular, minority groups defined by ethnicity and sexual identity are more anxious about genetic disclosures than other groups.

The rest of this paper summarizes in some detail over 60 articles published by GetPreCiSe researchers through the prism of these themes, specifically: 1) the nature of the risks posed by the collection of genetic data; 2) legal and scientific methods of minimizing those risks; 3) methods of safely increasing the scope of genetic databases; 4) public perceptions of genetic privacy; and 5) cultural depictions of genetic privacy. Together, they paint a complex, multifaceted portrait of how genetic privacy can technically be protected, the incompleteness of legal responses, and present and future views of what genomics may hold for humanity.

## 2 The risks posed by the collection of genetic data

Genetic data utilized in research projects is typically de-identified to protect the privacy of participants. However, certain types of attacks on this research, especially when the attacker already has some information about the target, presents the risk of re-identification, which allows genetic information to be matched to a known individual. GetPreCiSe research suggests that the risk of re-identification in properly maintained databases is at present not significant, but at the same time is non-trivial. Certain precautions, some outlined here and many more detailed in the next section, need to be taken to protect privacy.

In *Managing Re-Identification Risks While Providing Access to the All of Us Research Program*, Xia et al. (2023) examined the privacy risk associated with the All of Us Research Program, which makes individual-level genetic data available to researchers. The article focuses on how the data of the 329,084 people in the program was transformed to mitigate re-identification risk, including generalization of participants’ geographic regions, suppression of public events associated with collection that might be traced to an individual, and randomization of collection dates. The authors computed the re-identification risk for each participant using a state-of-the-art adversarial model assuming that the intruder knows that a particular person is a participant in the program. They concluded that the 95th percentile expected risk of re-identifying an All of Us participant is below the threshold used by various U.S. state and federal agencies. However, a subgroup analysis revealed that risk levels were higher for certain races, ethnicities and genders. The authors concluded by noting that, although the risk of re-identifying All of Us participants is low, the fact that risk exists justifies the program’s multipronged data protection strategy, which involves strong authentication practices, active monitoring of data misuse, and penalization mechanisms for users who violate terms of service.

Another article by Xia and colleagues, *Enabling Realistic Health Data Re-Identification Risk Assessment Through Adversarial Modeling* (Xia et al., 2021), points out that analysis of re-identification risk for biomedical data often assumes a worse case scenario, in which attackers know all identifiable features (e.g., gender, age, race) about a subject. Yet, worst-case adversarial modeling can overestimate risk and thus might induce unnecessary editing of shared data. This study introduces a framework for assessing the risk of re-identification that takes into account the attacker’s resources and capabilities, and concludes that, using that framework, the re-identification risk associated with attack scenarios based on the real-world capability is often significantly lower than under the worst-case assumption. This finding holds true for all case studies regardless of the size of the dataset and implies that substantially more data could be shared for biomedical research.

In *Re-Identification of Individuals in Genomic Datasets Using Public Face Images*, Venkatesaramani et al. (2021) study the privacy impact of developments that allow genomic data to be matched to human faces. The authors point out that such investigations assume access to well-curated images, which are rarely available in practice and are challenging to derive from photos not generated in a controlled laboratory setting. They find that, for most individuals, the actual risk posed by attacks that rely on linking genetic data to facial images is substantially smaller than claimed in prior investigations. Moreover, the authors show that such risk can be markedly reduced by adding a small amount of well-calibrated noise, imperceptible to humans. The results of this investigation create an opportunity to create image filters that enable individuals to have better control over re-identification risks that stem from attacks based on linking images to genetic data.

In *Building a Dossier on the Cheap: Integrating Distributed Personal Data Resources Under Cost Constraints* (Anindya et al., 2017), Imrul Chowdhury Anindya et al. raise the concern of re-identifying individuals through multiple collated datasets. Most prior work assumed an attacker would only be able to link a genetic dataset to one available data source, but the authors argue that the multi-billion dollar data broker industry opens new avenues for attack. While a genetic dataset and a target dataset may not share enough attributes for reliable record linkage–and, thus, would be considered protected under previous problem formulations–an attacker now may be able to construct a chain of intermediate connections between the two datasets by purchasing data from a broker. The authors model these connections mathematically using graph theory and develop an algorithm to identify the intermediate datasets that provide the highest chance of accurate linkage, subject to cost constraints on which datasets the attacker can purchase. The authors then demonstrate how such an attack may be conducted using actual voter registration data from two different U.S. states, as well as how to estimate the re-identification risk enabled by this type of attack.

Providing further insight into the importance of the attacker’s access to external data, Zhiyu Wan et al., in *Using Game Theory to Thwart Multistage Privacy Intrusions When Sharing Data* (Wan et al., 2021), use game theory to model responses to attacks in which the attacker has access to multiple databases (as might be true of some data-brokers). This work describes a re-identification scenario using a two-player Stackelberg (leader-follower) game in which one party acts and the other responds, in a scenario involving perfect information. The article uses this scenario as a basis for experiments with large-scale genomic datasets to show that, even assuming adversaries with the capability of launching multistage attacks, most data can be effectively shared with low re-identification risk. The authors also conclude that, based on these results, subjects should be more likely to share their data with an open data repository such as the Personal Genome Project if sharing partial data is permitted rather than prohibited (a result which may seem counterintuitive but suggests that participants want to take full advantage of the database, despite the privacy risk). This finding suggests that providing subjects with options could encourage a greater degree of data sharing without increasing the risk of re-identification. The authors further suggest that, to promote data sharing, policymakers could increase penalties for detected privacy breaches and data holders could increase rewards for data sharing.

GetPreCiSe research also suggests that the size of genetic databases should be taken into account when deciding whether or not to share one’s genetic data. In *When to Share Your Genome to an Online Community: A Game Theoretic Approach to Balance Privacy Risks and Familial Benefits*, Guo et al. (2023) used game theory to estimate people’s willingness to contribute genetic information to a DTC-GT company or third party interpreter, given the potential that law enforcement or others will discover familial relationships. Through a number of simulations using different assumptions about players’ general views on privacy, the authors found that for databases of sufficient size (which the authors estimate should involve about 3 million people), the possibility that a person’s genomic information will be discovered if they do not join a database is about the same as when they do join the database. In such a situation, the authors conclude the best strategy for potential users who seek to identify relatives or obtain some information about their own genome may be to contribute their genomic data.

In *Biomedical Research Cohort Membership Disclosure on Social Media*, Liu et al. (2019) investigated the dangers of self-disclosure—the practice of revealing on social media or elsewhere one’s participation in a genetic study. Researchers gathered and analyzed 4,020 tweets, over 100 of which disclosed the individual’s participation in a genetics research program. Approximately 80% of these tweets involved disclosure by the individual, with the remaining tweets involving disclosures by the program organizer (usually as a way of promoting the program). The authors concluded that disclosing one’s membership in a research cohort increases the chance that a person’s identity, genomic data, and other sensitive health information will be discoverable. Given this threat, research programs could inform participants about the risk of membership disclosure and make clear that if self-disclosures are made, privacy may not be guaranteed. Research programs should also inform participants of such threats when asking whether they can share information about participants (for example, through promotional stories). Alternatively, the program could consider sharing stories without mentioning the volunteer’s real name or quasi-identifiable information.

In *Detecting the Presence of an Individual in Phenotypic Summary Data*, Liu et al. (2018) report a study investigating the privacy risks associated with providing summary phenotypes (i.e., information indicating the interaction of genes with the environment). Other researchers had found that if an attacker has access to allele frequencies for 1) the study pool, 2) a reference population, and 3) the genomic variants of a targeted individual, then, under the right conditions, the attacker could predict with some certainty whether or not the target is a member of the pool. However, this study found that, as the number of phenotypic facts known to the adversary decreases and as the prior probability that a targeted individual is in the study pool rather than the reference set decreases (because, for instance, the reference set is much larger than the target set), the power of this attack diminishes and may be sufficiently low to support the dissemination of summary phenotypic information without amendment. However, this finding is based on the expectation that the adversary’s knowledge about the targeted individual and the reference dataset is limited. In the event that the adversary has greater knowledge, or the organization planning to share data has no information about the capabilities of the adversary, then additional safeguards may be needed. For instance, it may be more prudent for the organization to require users of the resource to enter into data-use agreements, suppress certain phenotypic variables that are driving the disparity between the pool and the reference, or employ some combination of the two (a strategy that has been shown to be feasible for the dissemination of genomic summary data).

In *Systematizing Genome Privacy Research: A Privacy-Enhancing Technologies Perspective*, Mittos et al. (2019) issue a caution about our ability to protect genetic privacy. Based in part on information they glean from a survey of 21 genomic privacy experts, they note that the efficacy of privacy enhancing technologies may be hindered by unique properties of the human genome. In particular, the fact that the sensitivity of genome data does not degrade over time may make long-term security protection difficult, given the possibility of advances in decryption methods in the future. Similarly, the authors suggest that the loss in scientific utility that stems from hiding data might be hard to counteract with existing cryptographic and/or differential privacy techniques.

**Key Findings**

• Estimates about the risk of re-identification of genomic data that are derived from attacks that assume adversaries already know a great deal about the target likely over-estimate real-world conditions. Overestimation of risks can induce unnecessary editing of shared data, making data repositories less useful.
• Nonetheless, the risk of re-identification is non-trivial, especially with the advent of databrokers that can piece together disparate pieces of personal information. Long-term security protection may also be difficult, given the possibility of advances in decryption methods.
• Other actions, such as a person’s disclosure that they are in a genetic database, may also increase risk.
• Several strategies (some of which are discussed in more detail below) may reduce the risk of re-identification and other undesired disclosures:
  ο The use of multipronged data protections strategies.
  ο Requiring users of a genetic database to enter into data-use agreements.
  ο Increasing penalties for privacy breaches.
  ο Cautioning participants in research cohorts not to publicize their membership on social media or in other ways.

## 3 Methods of reducing risks to genetic privacy

The research reported to this point indicates that the collection and sharing of de-identified genomic information poses a small but non-trivial risk of unauthorized disclosure and re-identification. But many of the articles in the previous section also suggested ways that this risk can be substantially mitigated. The research described in this section elaborates on a number of different legal and scientific strategies that can be employed to protect against the risk of re-identification while adequately informing participants of the potential risks to privacy. Although the two subsections below divide the discussion of privacy protections between regulatory and scientific approaches, in practice the two methods are often entangled, and many of the articles described here examine both avenues. Both sections also reveal that considerably more regulation is necessary to ensure genetic privacy is protected and that genetic science is pursued in an optimal manner.

### 3.1 Regulatory/legal methods of reducing risks

In *The Law of Genetic Privacy: Applications, Implications, and Limitations*, Clayton et al. (2019) examine the landscape of genetic privacy law in 2019, with a focus on federal statutes and regulations, including the Health Insurance Portability and Accountability Act (HIPAA) and the Genetic Information Nondiscrimination Act (GINA). They conclude that few, if any, applicable legal doctrines or enactments provide adequate protection against, or meaningful individual control over, disclosures of genetic information about individuals who are subjects of genetic research. While under these statutes patients are asked to sign an acknowledgement of a covered entity’s notice of privacy practices and thus may believe that they control their data and that their data cannot be used to cause them harm, the law’s protection is illusory in both respects. For example, the HIPAA Privacy Rule has numerous exceptions permitting third party access to individually identifiable health information. Access to the results of direct-to-consumer genetic testing is only cursorily regulated. The authors argue that it may be time to shift attention from attempting to control access to genetic information to considering the more challenging question of how these data can be used and under what conditions, explicitly addressing trade-offs between individual and social goods in numerous applications. They also suggest that these considerations are just as applicable to general health information as they are to genetic information.

In *Me and My Data*, Igo (2018) points out that, as an historical matter, personal data—whether collected for scientific, commercial or bureaucratic reasons—was often treated as confidential and protected, but at the same time was not typically thought about in terms of individual ownership. Starting in the late 1960s, however, more and more people in the industrialized West questioned who should be able to access information about individuals–the people themselves or the authorities who collected the data. The question was sparked as much by political and economic developments as it was by scientific and technological ones. Citizens’ moves to shore up their property claims prompted new regulations around access, control, and consent that continue to undergird contemporary ideas about personal data. A product of social movements and civil rights reforms as well as market thinking, this bid for authority over one’s “own” information would reveal its limitations by the turn of the 21st century in the context of big data economy, given the difficulty of, and lack of commitment to, fully implementing informed consent.

Echoing this theme, in *Assessing Risks to Privacy in Biospecimen Research* (Clayton et al., 2017), Ellen Wright Clayton and Bradley Malin critique the Office for Human Research Protections’ proposal to define biospecimens as human subjects regardless of their identifiability and so require express, elaborate informed consent for all uses. In a two-part analysis, the authors address whether this level of biospecimen exceptionalism is warranted empirically, legally, and as a matter of policy. They argue that the federal government’s proposed recommendations would unduly limit research by placing too much emphasis on remote risk and that they should instead reflect the actual probability that a person will be identified from genomic data. Ultimately, the drafters of the proposed rule elected not to include de-identified biospecimens within the definition of human subjects. They did, however, adopt a requirement that the definition of identifiability be reexamined routinely and suggested that examination should start with the whole genome sequence. This approach, the authors argue, can be understood as “biospecimen exceptionalism delayed.” What is truly needed, they contend, is an assessment of the real risks of re-identification and the development of strategies to mitigate those risks.

In *Dynamic-Informed Consent: A Potential Solution for Ethical Dilemmas in Population Sequencing Initiatives*, Dankar et al. (2020) investigate how informed consent requirements can be defined to encourage ethical participation in genetic data collection. They note that, while the majority of population-level genome sequencing initiatives claim to follow the principles of informed consent, the specific requirements for consent have not been well-defined in this context and in fact differ greatly across various initiatives, spanning, among other strategies, mandates for broad consent, blanket consent, and tiered consents. The authors propose the strategy of “dynamic consent,” which requires a personalized communication platform that supports continuous two-way communication between researchers and participants. The paper analyzes various current implementations of dynamic consent and assesses whether they enable participants to make autonomous and informed choices on whether or not to participate in research projects. They conclude that dynamic consent can facilitate an ethical long-term relationship with participants as serious partners in decisions related to their data. However, they also note major hurdles to overcome, as only one out of the five studies about dynamic consent reported in the article made an explicit attempt either to examine the comprehension of their participants or to evaluate the extent to which patient autonomy is facilitated. The authors suggest that blockchain technology that records and manages all data access may help reinforce participants’ autonomy because the decentralized nature of the technology removes the reliance on biobanks’ and grants participants’ complete control over their consent data, as well as over its modification and withdrawal, making them the real data owners. However, here too there are challenges, particularly those that arise from the broadcast property of blockchains, which may leak sensitive facts about participants.

In *Sociotechnical Safeguards for Genomic Data Privacy*, Wan et al. (2022) note that providing appropriate levels of privacy for genomic data will require a combination of legal and technical approaches that consider the context in which the data are applied. For instance, they point out that, by itself, giving individuals and groups granular control over genomic data that pertain to them—a practice endorsed by some commentators, particularly from indigenous communities (Islam et al., 2024; Yracheta et al., 2024, Budin-Ljosne et al., 2017)—risks reifying unwarranted fear of genomics and is likely to disrupt a wide array of advances in ways that do not align with public or local priorities. What may be needed is a combination of notice and some choice, accountable oversight of uses, and real penalties (both economic and reputational) for inflicting harm on individuals and groups. The authors suggest creating secure databases for specific purposes, with privacy-protecting tools and rules governing individual choice for inclusion that are appropriate for each, which can take the form of a well-structured law as well as private ordering using tools such as data agreements.

In *Protecting Research Data of Publicly Revealing Participants*, McKibbin et al. (2021) agree that, at present, re-identification seldom visits significant harm on an individual, even for those who disclose their participation in a data collection program. But the authors also express concern that the re-identification risk could increase as the depth and breadth of data grow. They argue that the law’s defenses to this problem are incomplete and recommend three regulatory steps: 1) potential participants should be informed of the implications of revelation so they can make informed choices about taking part and talking about their involvement in research; 2) holders of the data must put systems of oversight and public accountability in place and devise mechanisms to evaluate users and inform them of their obligations to respect the interests of participants; and 3) researchers using the data should adhere to these requirements as a threshold for continued data access, but should also be aware that they may well face liability as jurisdictions increase efforts to regulate data use and protect privacy.

In *Does the Law Require Reinterpretation and Return of Revised Genomic Results* Clayton et al. (2021) discuss the state of the law regarding the reinterpretation of genomic tests that were conducted because of the patient’s family history or current condition. These reinterpretations, which may eventually become standard clinical practice, could provide additional information or even information that conflicts with previous analysis. The authors note that, in contrast to self-enforced ethical norms, which some argue warrant returning results under some circumstances, the law relies on damages and penalties, imposition of which usually depends on what is reasonable considering the costs entailed and other potentially conflicting obligations. At the time of publication, there were no cases, statutes, or regulations that support a legal duty to reinterpret genomic tests or to report the outcomes of such reinterpretations. The authors suggest that physicians and labs, working with professional organizations, think seriously about how to ensure that patients receive and understand the new results and that policymakers clarify the legal consequences of not providing such information.

In *Ethical Issues in Genetics and Infectious Diseases Research: An Interdisciplinary Expert Review*, Walker et al. (2021) identified and systemized the unique issues at the intersection of genetics with public health regulations and the control of infectious diseases (ID), based on a review of the views of 20 experts in public health, law and genomics, biobanking, genetic epidemiology, ID medicine and public health, philosophy, ethics and ID, ethics and genomics, and law and ID. The authors asked the experts to focus on the collection, storage, and sharing of genetic information relating to ID. The exercise highlighted the risks associated with 1) reporting ID information to government authorities that might share it with other agencies, 2) informing tested patients of potentially over-estimated risks, and 3) using genetic predispositions as a way of triaging treatments that might privilege some groups over others. They argue that these areas require norms and policies to protect privacy and advance equity that go beyond current protections.

In *It is All in the Timing: Calibrating Temporal Penalties for Biomedical Data Sharing*, Xia et al. (2018) critique the various initiatives to make biomedical datasets more widely available to recipients who sign Data Use Certificate agreements. While these agreements often revoke the recipient’s data usage rights when violations occur, the revocation often lasts only a few months, based on the assumption that the scientific value of biomedical research data depreciates significantly after a short period of time. However, no studies have investigated this assumption. The study reported in this article finds that the impact factors for publications based on genetic datasets do in fact depreciate in a statistically significant manner, but slowly at a rate of only around 10% per year on average. Accordingly, the authors argue that revoking usage for short periods of time may not sufficiently deter those who would violate Data Use Certificate agreements and that alternative, longer-lasting, penalty mechanisms may need to be developed and implemented.

In *Registered Access: Authorizing Data Access*, Dyke et al. (2018) address how to make genetic information more widely available to researchers and clinicians. The authors describe the Global Alliance for Genomics and Health (GA4GH) data access policy model called “registered access,” which requires registrants to agree to basic terms and conditions about the use of DNA sequence and health data in research. This policy would enable a range of professionals, starting with researchers and clinical practitioners, to use and reuse data across multiple datasets within the bounds of consent restrictions and other ethical obligations. Based on a number of pilot Scientific Demonstration data sharing projects, the article provides additional ethical, policy, and technical guidance to facilitate the implementation of this access model in an international setting. The extent to which the registered access policy model is recognized and supported by stakeholders, including research ethics boards and the research community, will determine the resources the system will ultimately provide.

Turning to the topic of commercial testing, James Hazel and Christopher Slobogin in *Who Knows What, and When? A Survey of the Privacy Policies Proffered by U.S. Direct-to-Consumer Genetic Testing Companies* (Hazel and Slobogin, 2018) discuss regulation of DTC-GT companies, which have proliferated into the hundreds in the past several years. Based on an analysis of genetic material submitted by consumers, these companies offer a wide array of services, ranging from providing information about health and ancestry to identification of surreptitiously gathered biological material sent in by suspicious spouses. Federal and state laws are ambiguous about the types of disclosures these companies must make concerning how the genetic information they obtained is collected, used, and shared. This article reports a survey of the privacy policies these companies purport to follow. It canvasses ninety DTC-GT companies operating in the United States and provides a detailed analysis of whether and to what extent those policies inform consumers about how their genetic information will be used and secured, with whom it will be shared, and a host of other issues. Using the Federal Trade Commission’s articulation of the Fair Information Practice Principles and the agency’s proposed Privacy Framework as the baseline, they conclude that most policies fall well short of the ideal.

In *A World of Difference? Law Enforcement, Genetic Data, and the Fourth Amendment*, Hazel and Slobogin (2020) analyze constitutional and other restrictions on law enforcement use of genetic databases, especially those maintained by DTC-GT companies for law enforcement purposes. Police are increasingly requesting the DNA of an identified suspect from a database, or, more commonly, try to match crime-scene DNA with DNA profiles in a database to identify a suspect or family member of a suspect. Although both efforts would probably be called “searches” under common parlance, until recently they were clearly not searches for Fourth Amendment purposes because the Supreme Court has defined the word in terms of “expectations of privacy society is prepared to recognize as reasonable” and has construed that phrase narrowly, without reference to society’s actual views. This article reports an empirical study that attempts to gauge those views and finds that the Court’s decisions are antithetical to societal expectations as they apply in the genetic investigation context. The research also suggests that the location of genetic information, rather than its nature or the purpose for which it is acquired, is the primary driver of these intrusiveness ratings. For example, DNA given to a doctor for analysis is more closely associated with privacy than DNA given to a genealogy service or research entity. Based on this research, they argue that both police access to non-governmental genetic databases and police use of covert methods to collect DNA in the hope of matching crime scene DNA require judicial authorization, though not necessarily a traditional warrant. More broadly, they argue that empirical data about the public’s privacy concerns surrounding law enforcement’s collection of and access to genetic data should be an integral consideration in judicial determinations of how these activities should be regulated by the Constitution.

**Key Findings**

- Legal protections, such as those provided by the Health Insurance Portability and Accountability Act (HIPAA), the Genetic Information Nondiscrimination Act (GINA), and the Fourth Amendment’s restrictions on unwarranted searches are inadequate.
- Existing legal regimes all recognize numerous exceptions that permit third party access to identifiable health information.
- Policies of DTC-GT companies are often woefully inadequate at preventing dissemination of genetic data.
- Searches of DNA profiles in third-party databases should require judicial authorization as a constitutional matter.
- Potential participants should be informed not only of the non-negligible risks of re-identification but also of the potential for third-party access and data-sharing across companies and government entities.
- Efforts to assert individual control over one’s data have proven to be of limited utility in protecting genetic privacy.
- Although it is generally recognized that some form of consent for the collection and use of genetic data is important, the precise nature of that consent, which can range from dynamic consent to simple assent, is highly contested, and in fact the law allows many uses of genetic data without consent.
- Creating secure databases for specific purposes and tailoring privacy-protecting tools and rules that are appropriate for each situation may be superior to granting granular control over data in larger, general-purpose databases.

### 3.2 Scientific methods of reducing risks

In *A Framework for Privacy-Preserving Genomic Data Analysis Using Trusted Execution Environments*, Asvadishirehjini et al. (2020) argue that concerns about genetic privacy can be addressed through what they call “trusted execution environments” (TEEs), which guarantee that the execution of source code and the data under investigation remain in an isolated tamper-resistant chip. These TEEs provide confidentiality for sensitive data as well as a means of attesting to a program’s correctness. However, the authors argue, TEE protection can and should be further enhanced by using the same data-oblivious access patterns as those used by genome-wide association studies and deep learning genomic analyses.

In *Defending Against Membership Inference Attacks on Beacon Services*, Venkatesaramani et al. (2023a) address the advent of Beacon services, which were developed to broaden accessibility to genomic data by enabling users to query for the presence of a particular minor allele in a dataset; that information, in turn, helps care providers determine if genomic variation is spurious or has some known clinical indication. However, various studies have shown that this process can leak information that allows identification of individuals in the underlying dataset. There are various approaches to mitigating this vulnerability, but they are limited in that they typically rely on heuristics to add noise to the Beacon responses, offer probabilistic privacy guarantees only, neglecting data utility, and assume a batch setting where all queries arrive a once, an overly restrictive and unrealistic model for beacon data sharing. This article introduces principled algorithms that guarantee both privacy and, in some cases, worst-case utility in a Beacon service setting. Moreover, through extensive experiments, the authors show that the proposed approaches significantly outperform the state of the art in terms of privacy and utility, using a dataset consisting of 800 individuals and 1.3 million single nucleotide variants.

In a related piece, *Enabling Trade-offs in Privacy and Utility in Genomic Data Beacons and Summary Statistics,* Venkatesaramani et al. (2023b) and colleagues introduce optimization-based approaches that facilitate tradeoffs between protecting privacy and ensuring the utility of summary data or Beacon responses in the face of membership-inference attacks (which are designed to determine whether a person is part of a study associated with a particular disease). The authors consider two attack models based on likelihood-ratio tests: one where the attacker uses a fixed threshold and one where the attacker adaptively adjusts the linkage threshold to counteract privacy-protecting measures. The authors present a simple, scalable protection algorithm for both threats and demonstrate through an extensive evaluation with public datasets that their approach outperforms current state-of-the-art methods for protecting both utility and privacy, particularly in the context of the adaptive threshold attacker.

In *Controlling the Signal: Practical Privacy Protection of Genomic Data Sharing through Beacon Services*, Wan et al. (2017a) also address the vulnerability of Beacon services used by the Global Alliance for Genomics and Health (GA4GH). The authors describe the winning solution to a challenge hosted by the Integrating Data for Analysis, Anonymization, and Sharing (iDASH) Center designed to thwart the membership inference attack described by Shringarpure and Bustamante (Shringarpure and Bustamante, 2015). Wan et al. generalized the original challenge to make it representative of real-world scenarios and assessed the utility of the model in different conditions. They found that their method is more protective than other known methods and can be extended to address situations where the data custodian knows the query sequence of the attacker. The authors conclude that it is possible to thwart attacks on Beacon without substantially altering the utility of the system using their computational methods.

In *A Community Effort to Protect Genomic Data Sharing, Collaboration, and Outsourcing*, Wang et al. (2017) report on and analyze the impact of the third Critical Assessment of Data Privacy and Protection competition in 2016. This competition was a community effort to bring together biomedical informaticists, computer privacy and security researchers, and ethical, legal and social policy scholars to assess the latest advances in privacy-preserving techniques for protecting human genomic data. Teams were asked to develop novel protection methods for emerging genome privacy challenges in three scenarios: data sharing through the Beacon service of the GA4GH; collaborative discovery of similar genomes between two institutions; and data outsourcing to public cloud services. The outcomes suggest that applying highly optimized privacy-preserving and secure computation techniques to safeguard genomic data sharing and analysis is useful but that further efforts are needed to refine the techniques into practical solutions.

In *An Open-Source Tool for Game Theoretic Health Data De-Identification* (Prasser et al., 2018), Fabian Prasser et al. propose and evaluate the integration of game theoretic adversary analysis into a widely used de-identification software package. Game theoretic adversarial models allow for more data sharing (i.e., higher utility) than worst-case adversarial models at the same risk level, but game theoretic adversary models had yet to be implemented in de-identification software. In this work, the authors demonstrate how a game theoretic risk model can be used to guide common dataset de-identification techniques like domain generalization and record suppression in ARX de-identification software. They validate their method by analyzing 30,000 records from the U.S. Census under a variety of attack and payout conditions, compare their results to HIPAA Safe Harbor de-identification guidelines, and conclude that their method is scalable to genomic data.

In *Expanding Access to Large-Scale Genomic Data While Promoting Privacy: A Game Theoretic Approach*, Wan et al. (2017b) argue that legal deterrents such as data use agreements or strategies involving suppression or adding noise to genomic variants may unnecessarily impede research. As an alternative, they describe a game theoretic approach to develop more effective, quantifiable protections for genomic data sharing. They argue that their approach is fundamentally different from legal deterrents because it accounts for adversarial behavior and capabilities and because it tailors protections to anticipated recipients with reasonable resources, not adversaries with unlimited means. They also argue that this framework is applicable to other genomic data collection and sharing endeavors.

In a follow-up piece titled *Game Theory for Privacy-Preserving Sharing of Genomic Data*, Wan et al. (2020) expand on their contention that game theory can model rational interactions among the parties in a way that optimally balances utility and risks in genomic data-sharing scenarios. The authors first review recent advances in genomic data privacy that rely on game-theoretic approaches and characterize their core contributions as well as their limitations. They then report the results of two genomic privacy games: 1) the kinship game, where players of the game are from the same family, and 2) a game in which the data publisher and data recipient are players in the game. Based on their results, the authors propose models to help decision-makers choose how to optimally protect the privacy of their genomic data, while still helping medical research benefiting from the merits of genomics.

**Key Findings**

• Conventional assumptions about privacy risks can lead to situations in which information systems managing personal genomic data are grossly overprotected (and thus underutilized), but also grossly underprotected, leaving the data more vulnerable to misuse than anticipated.
• There are a number of quantitative and computational methods that can 1) determine the risk of re-identification for genomic data devoid of direct identifiers; 2) mitigate the risk to acceptable levels; and 3) help inform responsible genomic data management.
• These methods include trusted execution agreements, various types of algorithmic approaches, and models based on game theory.

## 4 Expanding the scope of genetic databases

Some commentators who are concerned about abuses of minoritized groups (Wallace, 2022) and inappropriate exercise of power by researchers (Jasanoff, 2022)—perspectives that have explored in depth by the Center for the Ethics of Indigenous Genomic Research (Center for the Ethics of Indigenous Genomic Research)—believe that expansion of genetic databases is an inappropriate goal. But to many others the benefits of such databases for medical and other valuable purposes probably outweigh the privacy costs associated with potential unauthorized disclosure, especially if one agrees with the demonstrations in the previously described articles on the limited nature of many privacy risks and the multiple ways of preventing them. Additionally, genetic databases to date have often failed to include individuals from all demographic groups, thereby compromising biomedicine’s ability to help all patients. The research below discusses ways to improve contributions to and access to genetic data, and also addresses ethical concerns of justice, representativeness, and discrimination in the course of collecting such data.

In *What Will “All of Us” Mean for Each of Us*? (Clayton, 2018a), Ellen Wright Clayton argues that to better understand how the interaction of factors such as ancestral origin, the environment, and stages of life affect human health and disease, enormous amounts of well-characterized data about individuals need to be collected and analyzed. She describes the “All of Us” program created by Congress as part of the 21st Century Cures Act to recruit more than one million participants willing to provide genomic, medical, behavioral, and exposure information over a several year period. She notes, however, that the promise of precision medicine to improve individual health is likely beyond the reach of many people for the foreseeable future for a number of reasons. For instance, most clinicians are ill-prepared to use this new genomic knowledge, and there are not enough genetics-informed personnel available to meet patients’ needs. Coupled with a lack of third-party insurance coverage for tests and follow-up care needed, the deficient knowledge base and resources in the field creates gaps that will be difficult to overcome.

In *A Representativeness-Informed Model for Research Record Selection from Electronic Medical Record Systems*, Borza et al. (2022) point out that scientific and clinical studies have a long history of inadequate recruitment of underprivileged and minority populations. This underrepresentation has led to inaccurate, inapplicable, and non-generalizable results. For instance, random selection from electronic medical record (EMR) systems, which now drives much research, often poorly represents these groups. Using information theoretic measures and an algorithmic approach, the authors introduce a method for quantifying representativeness. They apply this method to select cohorts of 2,000 to 20,000 records from a large (two million-plus records) EMR database at the Vanderbilt University Medical Center and assess representativeness based on age, ethnicity, race, and gender. Compared to random selection—which will on average mirror the EMR database’s demographics—they find that an approach adjusted for representativeness can compose a cohort of records that is approximately 5.8 times more informative for the general population.

In *Is It Time for a Universal Genetic Forensic Database*, Hazel et al. (2018) note that, while DNA is an increasingly useful crime-solving tool, it is still unclear how extensively law enforcement should be able to obtain genetic data housed in public and private databases. How one answers that question might vary substantially depending on the source of the data. Several countries have toyed with creating a “universal” DNA database, populated with data from every individual in society, obviating the need for any other DNA source. The authors argue that, although this move would be controversial, it may not be as dramatic as many think. In the United States, for example, the combination of state and federal databases (containing the genetic data of tens of millions of patients, consumers, and research participants) already provides the government with potential access to genetic information that can be linked to a large segment of the country, either directly or through a relative. But making the database truly universal would have several advantages. First, it would be less discriminatory because DNA will be collected from everyone, not just from individuals who have been charged or convicted with a crime (which is the primary practice today and disproportionately affects people of color). Second, it would eliminate the practice of using partial genetic matches to solve crime that today burdens innocent people who happen to be related to criminals and whom police may haphazardly treat as suspects. Additionally, if law enforcement agencies have access to a universal database, they will not be incentivized to use public direct-to-consumer sources or research databases for their investigation, which could enhance research by reducing public concerns that their data submitted for research will be misused by the government. If correctly implemented, a universal database would likely be more productive and less discriminatory than our current system, without compromising as much privacy.

In a counterpoint to the urge to take full advantage of genetic science, in *A Genetically Augmented Future* (Clayton, 2018b), Ellen Wright Clayton explores the possibilities of gene therapy and genetic enhancement. She argues that concerns about equity should lead society to develop guidelines for gene therapy to avoid a nightmare future in which a group of privileged people becomes stronger, smarter, and more beautiful than the rest. But because drawing lines between treatment and enhancement is difficult, the more likely and more unsettling scenario is that physicians will be left to rely on their own ethical commitments to decide when to use gene therapy.

**Key Findings**

• Properly carried out, expansion of genetic databases can be a boon to researchers without inappropriately infringing privacy. However, the promise of precision medicine—one goal of the All of Us project—will likely be beyond the reach of many people for the foreseeable future until enough clinicians are trained to use this genomic knowledge and coverage for care is enhanced.
• Inadequate recruitment of underprivileged and minority populations has limited the representativeness of databases, and thus has limited the benefit of genetic research for those populations. But computational methods of addressing representativeness can ameliorate this problem.
• In contrast to the makeup of research databases, people of color are *over*-represented in state and federal DNA databases associated with law enforcement. A universal genetic forensic database would be less discriminatory than our current system and can be regulated to avoid abuses.
• Concerns about equity should lead society to develop guidelines for gene therapy and genetic enhancement to avoid a future in which a group of privileged people becomes stronger, smarter, and more beautiful than the rest.

## 5 Public attitudes toward genetic privacy

Much of the concern about genetic privacy rests on the assumption that the public is particularly bothered by disclosures of genetic information about them. A number of GetPreCiSe articles suggest that this assumption does not hold true, at least in all situations or for all groups. Perceptions about privacy appear to vary significantly depending on the context and the person.

In *A Systematic Literature Review of Individual’s Perspectives on Privacy and Genetic Information in the United States*, Clayton et al. (2018) analyzed 53 studies involving the perspectives of over 47,000 participants on real or hypothetical privacy issues related to human genetic data. When asked specifically “are you worried about genetic privacy?” the general public, patients, and professionals frequently said yes. In many cases, however, that question was posed poorly or only in the most general terms. While many participants expressed concern that genomic and medical information would be revealed to others, respondents frequently seemed to conflate privacy with several analytically (and pragmatically) distinct concepts: confidentiality, control, and security. In response to more specific probes, people varied widely in how much control they wanted over the use of data. They typically were more concerned about use by employers, insurers, and the government than they were about access by researchers and commercial entities. Additionally, people were often willing to give up some privacy to obtain other goods, such as healthcare or finding relatives. Importantly, existing studies paid little attention to understanding the factors—sociocultural, relational, and media—that influence people’s opinions and decisions. The authors suggest that future investigations should explore in greater depth which concerns about genetic privacy are most salient to people and the social forces and contexts that influence those perceptions, as well as the social practices that will lead people to set aside worries and decide to participate in research.

In *Risk, Trust, and Altruism in Genetic Data Sharing* (Samad et al., 2023), Zeeshan Samad et al. ask the question: How does concern about genetic data privacy compare with other privacy concerns? The authors conducted behavioral economic experiments comparing risk attitudes about sharing genetic data with a healthcare provider to risk attitudes about sharing financial data with a money manager. Both scenarios involved identical decisions and monetary stakes, permitting the research to focus on how the framing of data sharing influences attitudes. To delve deeper into individual motivations to share data, the authors also investigated how data sharers’ altruism and trust affected their decisions. The findings (with 162 subjects) indicated that individuals are more willing to risk a loss of privacy of genetic data (for an anticipated return framed as health benefits) than they are to risk loss of financial data (for an anticipated return in financial benefits). They also found that 50%–60% of data recipients chose to protect another person’s data, with no significant differences between the genetic and financial frames. Functionally, the research suggests that individuals prioritize privacy in their finances over privacy of their genetic data.

In *LGBTQ+ Perspectives on Conducting Genomic Research on Sexual Orientation and Gender Identity*, Hammack-Aviran et al. (2022) conducted in-depth, semi-structured interviews in 2020 with LGBTQ+ identified individuals (n = 31) to explore the range of LGBTQ+ perspectives on genomic research using either sexual orientation or gender identity data. Most interviewees presumed that research would confirm genetic contributions to sexual orientation and gender identity. The primary reasons for wanting such confirmation included validating LGBTQ+ identities, improved access to and quality of healthcare and other resources, and increased acceptance in familial, socio-cultural, and political environments. Areas of concern included threats of pathologizing and medicalizing LGBTQ+ identities and experiences, undermining reproductive rights, invidious gatekeeping of health or social systems, and malicious testing or misuse of genetic results, particularly for LGBTQ+ youth. Overall, interviewees at that time were divided on the acceptability of genomic research investigating genetic contributions to sexual orientation and gender identity. Participants emphasized researcher’s ethical obligations to LGBTQ+ individuals and endorsed engagement with LGBTQ+ communities throughout all aspects of genomic research using sexual orientation and gender identity data.

In *The Public Perception of the #GeneEditedBabies Event Across Multiple Social Media Platforms: Observational Study* (Ni et al., 2022), Congning Ni et al. examined web-based posts on Twitter, Sina Weibo, Reddit, and YouTube about the #GeneEditedBabies event (involving genetic manipulations at birth that purported to provide lifetime immunity from the HIV virus). The authors found that, although almost all experts opposed this event, many who wrote web-based posts supported it. The primary reason for opposition was rooted in ethical concerns, whereas the primary reason for supporting gene editing was the expectation that such technology could prevent the occurrence of diseases in the future. This research provides evidence that posts on web-based platforms can offer insights into the public’s stance on gene editing techniques and also indicates that public attitudes often differ from the position of academics and policymakers.

In *Implicit Incentives Among Reddit Users to Prioritize Attention Over Privacy and Reveal Their Faces When Discussing Direct-to-Consumer Genetic Test Results: Topic and Attention Analysis*, Liu et al. (2022) investigate the face image sharing behavior of DTC-GT users in an online environment (Reddit), a study triggered by the observation that participants in the 23andMe subreddit were increasingly posting test results with face images that reveal ancestry composition and family reunion photos containing relatives discovered *via* DTC-GT. In their study, the authors found that, on average, posts including a face image received 60% more comments and had karma scores 2.4 times higher than other posts. Given this association between face image posting and a greater level of attention, they concluded that people appear to be willing to forgo privacy in exchange for attention from others. To mitigate this risk, platform organizers and moderators could inform users about the risk of posting face images in a direct, explicit manner in an effort to make clear that their privacy may be compromised if personal images are shared.

In a related piece, *Health and Kinship Matter: Learning about Direct-to-Consumer Genetic Testing User Experiences via Online Discussions* (Yin et al., 2020) Zhijun Yin et al. sought to better understand individual motivations and experiences in engaging with DTC-GT. Researchers collected 157,000 posts published by 16,500 Reddit users under the r/23 andme and r/AncestryDNA subreddits between 2013 and 2019. The data showed that posting rates increased sharply after popular promotional events—for example, Amazon Prime Day and Black Friday—and most posts were inquiries into, or status updates about, the testing process. In categorizing their findings, the authors found that two themes, which they characterized as Ancestral Origin (discussion of ethnic and demographic lineage) and Kinship/Feelings (discussion of identification of relatives), were the most frequently discussed (markedly ahead of, for instance, discussions of what might be learned about health risks). The theme of kinship exhibited the largest range of emotional response; some people became excited because testing allowed them to find their biological parents or other kin, while others became upset because they unexpectedly found that their parents or other kin were not biologically related to them. The research demonstrated that online social media platforms can serve as a rich resource for characterizing actual DTC-GT experiences, suggesting that consumers’ purchasing behavior is associated with societal events and that future investigations should consider how DTC-GT challenges our understanding of kinship structure and function, genomic privacy, and the interpretation of health risks.

In *Direct-to-Consumer Genetic Testing: Prospective User’s Attitudes Toward Information About Ancestry and Biological Relationships* (Hazel et al., 2021), James W. Hazel et al. reported six focus groups with a diverse sample of participants (n = 62) who were aware of but had not used DTC-GT in an effort to understand more about what people considering these tests thought about the potential value, risks, and benefits of such testing, especially when taking into account possible uses by third parties such as potential kin and law enforcement. Participants differed widely in the perceived value for their own lives of DTC-GT for ancestry and kinship information. Some perceived ancestry testing as a mere curiosity or entertainment, while others, particularly those who had gaps in their family history, few living relatives, or were adopted, saw greater value. Concerns about intrusion into one’s life by purported kin and control of data were common, with many participants expressing worry about secondary uses of data that could harm users or their families. The use of DTC-GT data for forensic genealogy elicited a particularly wide array of reactions, both spontaneously and in response to specific discussion prompts, mirroring the current public debate about law enforcement access to such data.

In *Public Attitudes Toward Direct-to-Consumer Genetic Testing* (Ruhl et al., 2019), Grayson Ruhl et al. used Amazon Mechanical Turk to administer a web-based survey to over 1,000 individuals to ascertain public attitudes about DTC-GT. Of the group, 20% had undergone testing and 30% said they were unlikely ever to do so, while 55% said they knew someone who had done so. Close to 50% were very concerned that DTC-GT companies would not protect their identity, but 63.0% felt comfortable sharing results with academic institutions, and 36.2% were fine with sharing results with third party companies that are required to provide the same privacy protections as the DTC-GT company [a level of protection that is highly variable, as our study “*Who Knows What, and When?*” showed (Hazel and Slobogin, 2018)]. Only 14.3% felt comfortable sharing their information with the government (unless used to solve a crime, in which case over 70% expressed little concern), and only 6.1% were willing to share with commercial entities like Google or IBM.

**Key Findings**

• Existing studies often pose questions about public perceptions of genetic privacy poorly and in broad terms. Further investigation is necessary to determine which concerns about genetic privacy are most salient to people and which social forces and practices influence the public’s attitudes and decisions about participating in genetic research.
• The notion of “genetic exceptionalism”—positing that genetic privacy is more important than other types of privacy—does not hold true. Individuals appear to prioritize financial data privacy over genetic data privacy, and in social media contexts may even be willing to sacrifice genetic privacy for attention.
• Web-based platforms can offer insights into the public’s stance on genetic technologies and DTC-GT companies, which often differs from the position of academics and policymakers, and can be heavily influenced by societal events and public debate.
• There is significant disagreement in the LGBTQ+ community about the acceptability of genomic research into the genetic contributions to sexual orientation and gender identity.

## 6 Genetic themes in American culture

Literature, film, television, social media, and popular culture are an important way to understand public perceptions of genetic privacy because their portrayal of genetic science furnishes the public debate with shared images, narratives, and possibilities for the future. In turn, advances in cutting-edge genetics influence the content of the popular media, as science opens up new possibilities for cinema and television to explore. To delve into these dynamics, numerous GetPreCiSe studies looked at films, medical dramas, superhero media, fandom, genetic “outsider” depictions, dystopian fiction, and other cultural sources.

In his book *Literature, Science, and Public Policy: From Darwin to Genomics* (Clayton, 2023), Jay Clayton urges readers to embrace the pragmatic function of literature: how literature has both reflected and helped shape public attitudes toward evolution and genetics. Hence, literary studies and the humanities can contribute to science policy, providing insights into the public’s understanding of genetics that are not available elsewhere and often cannot be uncovered by the research methods employed in the social sciences. The book charts the reciprocal exchange between literature and the life sciences across three distinct epochs: the late 19th century response to Darwin; the 1930s through the Cold War, when the modern synthesis of evolution and genetics was developed in context of modernity; and the 21st century, the age of genome time (where genes can help predict the future). This discussion of literature deepens and expands awareness of the many forces that constrain and enable us in the present genetic era.

In *Genetics in Film and TV, 1912–2020*, Gibbons et al. (2021) explore how genetic science and technology are presented on screen. The investigation was driven by three initial hypotheses. The first was that the portrayal of genetics in film and television would vary over time in parallel with highly publicized advances in genetic science as well as with broader historical developments. Second, variations in the constraints and opportunities presented by the different media—film v. television—would also create variation in the representation of genetics. Finally, different genre conventions would play a role in shaping the ways in which genetics is portrayed on screen, and these differences would be discernible from both the nature of the medium and the release date. Using a unique data set of 238 films and 537 television episodes, this article tracked the depictions of genetics in these forms of popular culture over more than a century. The authors found that the earliest part of the dataset frequently featured eugenics as a positive theme. After the decline of eugenic ideas post-World War II, eugenics reemerged as a theme in the 2010s, primarily in response to the possibility of genetically enhanced children, athletes, and soldiers. The genre of media also played a role: science fiction predominantly portrays genetics negatively, whereas shows centering around medicine and law enforcement are more likely to depict genetics positively. These results may both reflect and increase public concern over the long-term possibilities that genetic science will threaten the future—i.e., their children’s identity or their own privacy—while more immediate applications of advances in genetic medicine and law enforcement appear to be perceived more positively, surmises that are supported by our survey data on public attitudes reported above (Samad et al., 2023; Ruhl et al., 2019).

In *Time Considered as a Helix of Infinite Possibilities* (Clayton, 2021), Jay Clayton explores the temporal implications of genomics through the lens of a classic science fiction story by Samuel R. Delany, “Time Considered as a Helix of Semi-Precious Stones.” Delaney’s futuristic version of “hologramic information storage,” which allows the interplanetary Special Services to discover and predict everything a suspect has done or will be doing at any time in the past, present, or future, resembles “genome time,” the illusion that data encoded in your DNA that is knowable from a single test in the present can reveal your entire life—not only where you came from but what you will become. The temporal implications of genomics are compared with “queer time” and contrasted with the temporal implications of nanoscience and climate change as a means of clarifying what is distinctive about genome time. Some practical consequences of genome time are also discussed. For instance, despite the relatively high percentage of false positives in DTC-GT, the varying results from one company to another, and the protests of clinicians who are confronted with data about genetic probabilities they are poorly prepared to interpret, consumers continue to flock to DTC-GT companies for what they regard as information about their future, comparable to the concept of genome time in Delaney’s science fiction story.

In *“DNA Doesn’t Lie:” Genetic Essentialism and Determinism* (Lillydahl and Clayton, 2025), Alice Lillydahl and Jay Clayton identified and viewed 38 episodes from *Law and Order: Special Victim’s Unit*’s first twenty seasons that centered on genetic themes in forensic contexts. Two recurring themes emerged: 1) that the role DNA plays is only one factor in a complex web of biological and social considerations that shape our understanding of kinship; and 2) that genetic predispositions to behavioral traits such as mental illness or violence should not be seen as obscuring the responsibility of personal choice. Although *SVU* rejects genetic essentialism and determinism by portraying social factors as trumping biological factors, it does so by overemphasizing the difference between nature and nurture. The necessity of resolving the case at the end of each episode leads the latter to win out over the former, thus missing the opportunity to build a complex, nuanced understanding of identity and family that acknowledges the power of both.

In *Eugenics and Genetic Screening in Television Medical Dramas*, (Eilmus and Clayton, 2024), Ayden Eilmus and Jay Clayton evaluate the contrasting depictions in television medical dramas of reproductive genetic screening and eugenics—two medical themes that some commentators see as closely related. By conducting a content analysis of 32 episodes of doctor shows featuring eugenic and/or genetic screening themes, the researchers put the medical drama landscape in conversation with bioethics scholarship and mark a significant divergence between the two. Doctor shows tend to champion genetic screening as a powerful tool for promoting individual reproductive choice and criticize eugenics as a socially unjust infringement of reproductive freedom. In doing so, medical dramas mark a subtle but important moral distinction between the population-level implications of eugenics and the highly personal, emotional impact of genetic screening, a distinction that is not always given weight by those bioethicists who see genetic screening as a prequel to eugenics. These findings echo those of Congning Ni et al., which indicate that public attitudes about gene editing often diverge from the positions of academics and policymakers, particularly when novel technology is used to prevent disease (Ni et al., 2022).

In a related piece, *Genetics in Television Medical Dramas* (Furman and Clayton, 2021), Lauren Furman and Jay Clayton surveyed medical dramas that aired on U.S. television from 1961 to 2019. They found that medical dramas have evinced a comparatively positive attitude toward genetics over time, acting as one of the strongest advocates in popular culture for genetic testing and genetic medicine. While other genres in television, film, and fiction warn about the many imagined dangers that will result from advances in genetic science—horrific mutation, biological warfare, threatening clones, and social stratification—medical dramas tend to portray more favorable alternatives: effective diagnoses, research and development of new treatment for genetic disease, and gene therapies. While not all the episodes have entirely positive outcomes, the medical contributions of genetics are cast in an overarchingly positive light. Indeed, as the findings of Samad et al. that were described earlier demonstrate (Samad et al., 2023), individuals are more willing to risk a loss of genetic privacy for an anticipated return in health benefits than they are to risk other kinds of data privacy.

In *Genetic Privacy Breaches in Television Medical Dramas* (Furman, 2020), Lauren Furman focused on two episodes from the survey of medical dramas just described that portrayed instances of privacy breaches involving the DNA of patients in diametrically opposed ways. While one early episode emphasized the importance of respecting the patient’s right to privacy, the second episode was emblematic of more recent medical dramas that often excuse ethical breaches and exalt charismatic bad actors, a trend which may affect how the public views ethical questions around data privacy. Heavy consumers of media are likely to perceive fictional representations of complex subjects as accurate to real life, contributing to their expectations for their own medical care and understanding of how privacy operates in medicine.

In *Genetics in the X-Men Film Franchise: Mutants as Allegories of Difference*, Grimsted et al. (2023) analyzed the complete corpus of live-action X-Men movies, focusing on their depictions of genetics and otherness. The researchers watched and qualitatively coded all thirteen movies produced by 20th Century Fox that take place in the same shared cinematic universe, beginning with *X-Men* (2000) and ending with *The New Mutants* (2020). The X-Men movies are unusual summer blockbusters since they explore genetic topics through their central characters—mutants who are genetically different from their non-mutant peers. Mutants in the films evoke a plurality of analogies, such as mutant-as-Black and mutant-as-queer. These intersecting metaphors build upon a core of genetic difference to create a versatile but limited picture of prejudice, solidarity, and otherness.

In a related piece, *Germans and Genes on Screen: Marvel’s X-Men Films* (Porter, 2021), Cynthia Porter argues that the X-Men films show how an ostracized and oppressed group can become susceptible to additional abuse through forced medical experimentation, a concern about genetic harm echoed by survey data developed in other GetPrecise research (Hammack-Aviran et al., 2022). Further, the films show mutants undergoing experimental procedures designed to transform them into potential weapons for the benefit of the government. As a whole, the franchise explores the theme of weaponizing victims of oppression by forcing them to engage in activities ranging from military service to personal security details. An element that links the X-Men films with contemporary conversations around genetic privacy is the emotional, psychological, and physical manipulation of mutants presented in the films and how these ethical breaches relate to informed consent.

In *Monstrous Proletariat: The Racial Chimera in “District 9” and “Sorry to Bother You*” (Taylor and King, 2022), Terrell Taylor and Claire Sisco King focus on two films, Neil Blomkamp’s *District 9* (2009) and Boots Riley’s *Sorry to Bother You* (2018), that deploy spectacular renditions of bodily transformation as allegories of the violence of racial capitalism. The films portray malevolent corporations that transform human beings into non-human creatures (chimeras) in the pursuit of wealth, gesturing towards comparisons between the corporate dehumanization of today and various histories of violent dehumanization, including chattel slavery, the Holocaust, and Apartheid. The films make clear that public understandings (or misunderstandings) about genetics remain central to ongoing debates about race and racial justice. The films also demonstrate the power of the racial chimera in many of the binaries that govern so many conversations about genetics and race, including self/other, sameness/difference, human/nonhuman and species/race.

In *How Can Literary and Film Studies Contribute to Science Policy? The Case of Henrietta Lacks* (Clayton and King, 2020), Jay Clayton and Claire Sisco King discuss the impact of literature, film, and television on public concerns about the privacy of their medical data, using as a case study Rebecca Skloot’s bestseller, *The Immortal Life of Henrietta Lacks*, and Oprah Winfrey’s adaptation of the book, which the authors analyze both qualitatively and quantitatively. These two works demonstrate that the violation of a patient’s privacy can have devastating and far-reaching effects beyond the individual. It can affect relatives for generations, touch neighbors and friends, and influence attitudes of a still-wider community—in this case, African Americans in Baltimore, and beyond. The authors also propose a new model for literary scholars to contribute to large-scale, multiyear collaborative research on public policy that brings together science and humanities.

In *Race, Gender, and Genetic Privacy in Kay Jamison’s “An Unquiet Mind” and Meri Nana-Ama Danquah’s “Willow Weep for Me*” (Hagaman and Clayton, 2024) Sarah Hagaman and Jay Clayton analyze two memoirs from the 1990s of women struggling with hereditary mental illness who express anxiety about revealing their conditions and about whether their revelations will violate the privacy of their close relations: Kay Jamison’s *An Unquiet Mind* (1995) and Meri Nana-Ama Danquah’s *Willow Weep for Me* (1998). These memoirs reveal the gendered and racial barriers to authentic self-representation when disclosing hereditary mood disorders in clinical settings. Intersectional language in these memoirs allows women to give voice to their hereditary conditions that are harder (or unsafe) to voice to doctors and thus allow access to private identity on their own terms. In the process, these memoirs demonstrate that discussions of genetics in clinical settings about people with hereditary diseases carry risks of stigmatization, discrimination, and even eugenic pressures not to reproduce. These texts also reveal that mood disorders cannot be separated from questions of race, gender, and class, and they anticipate contemporary concerns about genetic privacy’s precarity across demographics. The authors argue that complex, intersectional notions of hereditary mood disorders must be central to understanding 21st century genetic privacy in future policy decisions.

In *Contested Kinship in Two Serial Comedy-Dramas, “Workin’ Moms” and “Living with Yourself*” (Hagaman, 2020), Sarah Hagaman examines two comedy-drama television series, *Workin’ Moms* (2017–2020) and *Living with Yourself* (2019–2020) that feature 21st-century twists on the problem of uncertain paternity. *Workin’ Moms* follows an unmarried same-sex couple who meet a man claiming to be the sperm donor for their infant son. *Living with Yourself*, in contrast, features a heteronormative married couple and a woman’s possible impregnation by her husband’s clone. Both shows depict the father’s formal contribution to the genetic makeup of a child as more important than the maternal and familial environment. The notion that the paternal genetic contribution is uppermost evokes discredited views of heritability that have persisted for centuries.

In *The Response to Female Characters in Genetic Films* (Lillydahl, 2020), Alice Lillydahl evaluates the roles provided for women actors in the fifty highest grossing films of the last 2 decades that feature genetic content. With one exception (*My Sister’s Keeper*), none of the fifty films in the study focus on the types of tropes that used to be called “women’s issues,” suggesting that popular genetic films tend to be male-dominated blockbusters. To investigate this issue further, the author matched women’s ratings of the fifty films in the Internet Movie Database (IMDb) with outcomes on the Bechdel Test (which asks, does the movie have two named female characters who talk to each other about something other than a man?) and the Mako Mori Test (which asks, does the movie have at least one female character who has her own narrative arc and is not supporting a man’s story?). The author found that women rated movies that did well on the latter test significantly higher on IMDb than movies that failed the test (while finding no relationship with Bechdel Test outcomes). The results reflect a desire from women to see independent and autonomous female characters.

In *What We Talk About When We Talk About Cloning: A Literature and Bioethics Perspective on Genetic Privacy, Consent, and the Family* (Hamann-Rose, 2021), Paul Hamann-Rose argues that a uniquely helpful perspective on the legal, ethical, and political uncertainties surrounding the complex of genetic privacy, consent, and the family can be gleaned from literary fictions about cloning. In Eva Hoffman’s novel *The Secret*, Hoffman’s contribution to bioethical debates about privacy through the figure of the clone is both allegorical, in that the anxiety evoked by cloning represents a similar anxiety aroused by genetic privacy, and literal, in that cloning inherently includes a use of genetic information to which the clone has not given consent. In *Never Let Me Go*, Kazuo Ishiguro explores the clone’s failure to combat their compromised genetic privacy. The novel serves as a reminder that one of the most dramatic privacy harms is not the initial infringement of privacy but the inability to do anything about its consequences. These two cloning narratives connect complex notions of privacy, consent, and family in the context of genetic research and around the changing forms of intimacy produced by biotechnology’s extension into the private sphere. These texts not only highlight genetic privacy as a pressing concern for bioethics scholars and science policymakers today but reveal the issue to have an emotive and psychological depth that crucially shapes its impact on society.

In *Under Surveillance: Genetic Privacy in Margaret Atwood’s MaddAddam Trilogy* (Hamann, 2019), Paul Hamann analyzes Margaret Atwood’s MaddAddam trilogy, the novels *Oryx and Crake* (2003), *The Year of the Flood* (2009) and *MaddAddam* (2013). Atwood’s trilogy registers a shift in conceptions of privacy from a focus on the violation of physical and psychological boundaries to concerns over the control of the use of personal data. In the novels’ pervasive surveillance culture, lacking control over genetic information limits individual agency and threatens a fundamental concept of modernity. The ethical concerns about risks to genetic privacy raised throughout Atwood’s trilogy are inextricably linked to questions of agency and power. The novels suggest privacy cannot be thought of in isolation from corporate and government surveillance, social divisions of wealth, and advances in science and technology, an assertion that is reflected in our group’s findings about concerns over employer, insurer, and government use of genetic data (Hazel and Slobogin, 2020; Clayton et al., 2018).

In *Reactions to the Direct-to-Consumer Genetic Testing Industry in Michael Connelly’s Fair Warning* (Tonkunas, 2020) Gabija Tonkunas discusses *Fair Warning*, a thriller novel involving the hunt for a serial killer who uses stolen DNA information from a fictional DTC-GT company to target his victims. The novel was published not long after the identification of the Golden State Killer *via* forensic genealogy from a direct-to-consumer genetic testing company to assist the police investigation. Reviewing comments made on the book’s Goodreads page, Tonkunas seeks to discover how popular fiction informs or shapes public attitudes towards DTC-GT and data privacy issues. Nearly a quarter of the reviews mentioned the need for regulation in the direct-to-consumer industry, demonstrating that the depiction of inadequate regulation in the fictional company leads readers to perceive actual risk in the real-life industry [which our research indicates is an accurate perception (Hazel and Slobogin, 2018)]. The novel’s fictional exposé of the poor regulatory oversight of genetic data privacy clearly resonated with the audience, encouraging many readers to think about the security of their own data and how to be responsible consumers. These anxieties are also reflected in the findings of Ruhl et al. reported earlier, which showed that 50% of survey participants were very concerned that DTC-GT companies would not protect their identities (Ruhl et al., 2019). The reviews analyzed by Tonkunas indicated that some potential customers even turned into advocates against these DTC services after reading the novel. Readers have absorbed the story, its characters, and its lessons as if the book were a convincing work of nonfiction rather than a thriller novel.

In *Queer Kinship: Privacy Concerns in Orphan Black* (Casey and Clayton, 2021), Marcie Casey and Jay Clayton examine the acclaimed Canadian television series *Orphan Black* (2013–2017), which they note poses a question straight out of the pages of science fiction: What would it be like to encounter multiple versions of yourself in the form of clones that you never knew you had? The authors highlight the potentially positive implications of novel kinship systems portrayed in *Orphan Black*, such as how they destabilize heteronormative restrictions on sociality. They also illuminate the difficulties and potentially negative side effects of such a system: the assimilation of new kinship structures on traditional familial norms; the difficulty of finding a language for reconstructing notions of sociality; the commodification and capitalization of queer bodies; the risk of new forms of eugenics; and the difficulty of obtaining user consent, given that the scope and implications of such future research is immeasurable.

In *Bioethics in “Orphan Black” Fan Fiction* (Eilmus, 2020), Ayden Eilmus analyzed the rich and understudied fan culture that has grown up around *Orphan Black*. Chief among the platforms through which viewers engage with the show is fan fiction (fan-written stories), an open access, de-monetized, and conversational medium especially well-suited to demonstrating what audiences think is important about a cultural form. The survey found that autonomy was the most prevalent bioethics theme in the fan fiction that builds on *Orphan Black*, followed by surveillance. The author points out that the generative and collaborative nature of fandom shifts the power to make decisions about what matters away from the producers of a television show and toward its audience, who then claim a degree of creative autonomy for themselves. Fan fiction writers utilize this agency to produce new interpretations of *Orphan Black* that are then shared and accepted across a large and diverse community, marking a novel process of meaning making. This increasingly common practice enables fans of *Orphan Black* to collectively shift their focus to the principle of autonomy within their own world, the show itself, and countless alternative universes.

In a related work, *Autonomy and Bioethics in Fan Responses to “Orphan Black*,” Eilmus et al. (2024) assess how fans of *Orphan Black* process the bioethical themes that are prominent in the show through an analysis of 182 viewer-created blog posts. Using a mixed methods approach, their findings reveal that *Orphan Black*’s fans distill the essence of the show down to its characters’ fight for autonomy. Furthermore, fan blogs reveal two notable pathways through which this bioethical principle is explored: gender and reproduction. Viewers draw strikingly emotional connections between the moral problems they observe on screen in *Orphan Black* and those they see in the real world—both today and in a possible future—particularly as those problems affect women. While existing scholarship acknowledges these themes in the show itself, the authors’ approach demonstrates science fiction fans’ active participation in meaning-making and bioethical reasoning and offers a novel approach to studying the public’s understanding of science and their affective response to it.

In *Genetics and Ethics in the “I am Legend” Corpus* (Feldman and Clayton, 2021), Zachary Feldman and Jay Clayton look at the potential dangers of genetic manipulation brought out in the three movies based on the novel, *I am Legend*. In all three films, a plague wipes out almost all of humankind but also creates undead vampires. While in the first two movies the plague is the result of external threats (a wind-born germ and chemical warfare), in the last film it results from genetic engineering by a scientist believing the virus is a cure for cancer. The authors argue the “I am Legend” stories reflect a shift from fears of external threats characteristic of contagions and warfare to concerns about dangers internal to twenty-first century science, particularly genetics. More specifically, they contend that the stories pinpoint several changes in the cultural response to biological threats: 1) they mark one sign of the emergence of genetics as a threat in the cultural imaginary; 2) they chart the movement of this threat from viral infection, spread by others, to the unintended consequences of genetic engineering; 3) they register the movement from external threats involving Othered groups to internal threats such as tampering with our genes; and 4) they explore the way popular media forms like science fiction and horror films often perpetuate cultural blind spots toward research on populations (i.e., the undead vampires) that have suffered civil death or have been reduced to what the philosopher Giorgio Agamben calls “bare life.”

In *The End of Genetic Privacy in the Blade Runner Canon*, Oliver et al. (2021) analyze how the narratives in the original Blade Runner novel and the two later movies test privacy limits differently over time. In the 1982 movie’s use of the Voight-Kampff empathy test, an invasive procedure that measures physiological reactions to a series of emotionally provocative questions, violates one’s privacy in physical and psychological terms. In the 2018 film by director Denis Villeneuve, *Blade Runner 2049*, the creation of genetic profiles has become standard practice. These profiles are registered at birth, barcoded, and exhaustively linked to one’s health data, personal activities, consumer behavior, and even memories. The progression of the canon mirrors real-world genetic and technological developments, parallelling today’s interconnectivity between an individual’s genetic data *via* DTC-GT services, consumer behavior, and health records. It thus both highlights the creeping acceptance of genetic privacy infringements in exchange for social and emotional fulfillment and depicts the bleak outcomes in a society that has systematically abdicated genetic privacy rights. In particular, Villeneuve’s *Blade Runner 2049* envisions a crushingly lonely future in which individuals are willing to give up privacy to fill their emotional void. The Blade Runner canon is a reflection of—and warning about—the shifting nature of our society’s concerns about genetic privacy.

In *Transdisciplinary Collaboration: Theory and Practice*, Clayton et al. (2025) summarize the scholarship of the GetPreCiSe group that focused on cultural issues and genetic privacy. They call this scholarship “transdisciplinary” because, in contrast to the more common interdisciplinary enterprise, it openly encourages researchers to pursue their own disciplinary approaches rather than by trying to synthesize different perspectives. The authors note that, while transdisciplinary research is rare in literary studies, it is well-illustrated by the two dozen articles published by the GetPreCiSe culture group, 13 of which were co-authored across disciplines. The authors summarize the four most important findings of the group’s qualitative and quantitative analysis of over 800 films, television shows, and novels as follows: 1) over time, conceptions of privacy have migrated from a physical “right to be let alone” toward a concern over how one’s private information is managed by institutions, corporations, and social media 2) privacy breaches, when they occur, profoundly affect families and communities, not just individuals being treated by doctors; 3) relatedly, the intersectional nature of privacy harms needs to be taken into account in privacy regulations and remedies, because violations of data privacy can result in far-reaching collateral damage to relatives and communities, especially for those living in poverty and subject to racial, sexual, and other forms of discrimination; and 4) the affective response to private information is fundamental to assessing public attitudes toward personal data, as demonstrated by fan outrage at various artistic depictions of genetic injustices that they saw encompassing problems in the real world.

**Key Findings**

• Literary studies and the humanities can contribute to genomic privacy policy by providing insights into the public’s understanding of and attitudes about the use and misuse of genetic data that will supplement research methods employed in the social sciences.
• Studies of viewer responses to popular media highlight the public’s active participation in genetic meaning-making and uniquely capture people’s emotional as well as intellectual responses to use of genetic data by physicians, researchers, private companies and the government.
• Portrayals of genetic privacy issues on screen and in literature—whether the genre is science fiction, historical depictions, or modern-day drama—often provide nuance to the issues involved.
• Prior to the 21st-century, genetic privacy issues depicted in film and TV emphasized a “right to be let alone”; in the new millennium, genetic privacy issues have often emphasized worries over how one’s private information is managed by institutions, corporations, and social media.
• Investigations of cultural media make clear that privacy breaches affect not only the individuals whose privacy is infringed but the person’s family and community, and that intersectional privacy harms are especially profound.

## 7 Conclusion

In a world in which genomic information is increasingly important to medical, epidemiological, and recreational pursuits, the work published by the GetPreCiSe Center over the past 9 years provides a transdisciplinary examination of genetic privacy that is both deep and wide-ranging. Of particular importance is GetPreCiSe’s examination of four areas: the privacy risks inherent in the collection, storage, and use of genetic information; legal and scientific means of limiting those risks; the need to expand genetic databases despite those risks; and society’s perception of the risks as reflected through samplings of both the public’s views and depictions in the arts. The conclusions reached in this research are nuanced and not always consistent with one another, but they provide a springboard for meaningful policy advances in the genetic privacy domain.

With respect to the privacy threats posed by the millions of genetic profiles that now reside in research and DTC repositories and increasingly in patients’ medical records, one principal focus of GetPreCiSe research was on the efficacy of de-identification and re-identification practices. The general consensus of that research is that the removal of crucial identifiers protects genetic data against most attempts to associate specific genetic information with specific individuals. However, increasing sophistication of such attacks, especially by entities with access to multiple databases and significant resources, presents a non-negligible risk of discovery, especially as the number of entities sharing the genetic information multiplies, the duration of genetic storage lengthens, and the attacker’s knowledge of the target’s characteristics increases (which is especially likely if the target self-discloses participation in a data collection program).

Accordingly, GetPreCiSe scholars, in accord with general best practice, recommend that holders of genetic information demand strong authentication of data users, inform data users of their obligations and potential liability, actively monitor and evaluate their use of the data, and penalize users who violate terms of service. Unfortunately, as several articles point out, formal legislative or judicial mandates fall far short in implementing these goals. The law also falls short with respect to protecting genetic information housed by DTC-GT companies which, as the fall-out from the bankruptcy of 23andMe demonstrates, (Linder, 2025), often fail to follow standard informed consent and privacy practices, may not demand sufficient authorization from the government when it seeks investigative material, and need to do more to protect against third party access. In part to deal with the legal vacuum (but also with the hope of stimulating specific legal regimes), several of the articles recommend private or technological protections, including: data use agreements between holders and users that revoke data usage privileges in cases of violation (perhaps for relatively long periods of time); suppression of certain phenotypic variables before transfer of information; trusted research environments that can transform data in a data-oblivious manner using secure software; and various algorithmic and game theoretic means of protecting Beacon and related services that go beyond traditional practices, limit the adverse impact on, and perhaps even enhance, the utility of the database.

Some disagreement surfaced in the GetPreCiSe corpus as to the viability of these practices over the long-term and in the face of concerted attacks. Further, while a few papers took the position that fully informed consent is necessary for every stage of the collection and use process, more papers argued that informing subjects of all risks, however remote, might not be possible or legally or morally required. Mandating fully informed, dynamic consent could also deter participation in valuable programs (thereby compromising representativeness) and could tempt researchers to be less careful in meeting their responsibilities in data management on the assumption that individuals had agreed to all possible uses. There are also outstanding questions about the extent to which subjects should be told of genetic testing research results, especially when they are tentative, as well as questions about the obligation to reinterpret these results over time and report the reinterpretation to participants.

In contrast, there was little dispute within the Center over the value of collecting genetic data if appropriate precautions are taken (although, as just noted, the nature of the preferred precautions varied). Virtually all of the GetPreCiSe papers assumed the value of large, properly protected genetic databases, at least for medical research purposes (and perhaps for properly regulated law enforcement purposes as well). Indeed, several papers focused on ways to expand both the scope of genetic databases and the ability of researchers to access them. A few papers paid particular attention to increasing the representativeness of these databases to ensure that all patients could benefit from the resulting research, while other papers noted that clinicians might not yet be ready to apply that research in a professional, ethical manner.

Not surprisingly, public and cultural attitudes about the importance of genetic privacy, the efficacy of protective measures, and genetic research are diverse and nuanced. Several GetPreCiSe articles directly sampled public opinion about disclosure of genetic information to employers, insurers, the government, DTC-GT companies, researchers, and doctors, with concerns about disclosure generally diminishing as one goes down that list (except, perhaps, for those who—like LGBTQ+ individuals or people with certain types of diseases—fear that even clinical professionals will let bigotry control their actions). GetPreCiSe research also found that, while genetic privacy is important to people, financial and other types of privacy may be just as important, if not more so, and that many people are willing to trade genetic privacy for health and other benefits, including benefits as ephemeral as gaining more attention on social media, suggesting that there is little or no “genetic exceptionalism” in the area of privacy. Similarly, GetPreCiSe survey research suggested that, despite expressing concern about DTC-GT breaches of privacy, much of the public was willing to use these companies to obtain ancestry and kinship information and tended not to be bothered by the prospect of academic institutions’ or law enforcement’s using those companies’ results for research or investigative purposes. While this relative lack of concern about genetic data may be changing in light of increasing access to data by government and by large language models, several of these studies suggested that privacy experts may underestimate the extent to which the public values utility over privacy risk.

The Center mined a diverse range of arts and popular culture for further insights into society’s views and found a similarly complicated picture about attitudes toward genetic privacy. Collecting and analyzing the largest dataset of films and TV shows about genetics ever assembled, GetPreCiSe researchers documented changes in the types of worries about privacy violations over time. Prior to the 1990s, concerns about genetic privacy mostly involved potential psychological and financial harms to individuals. In subsequent decades, as public attention focused on the sequencing of the human genome, popular culture came to emphasize population-wide harms resulting from the aggregation and sharing of data for commercial, medical, and law enforcement purposes. Science fiction and horror, in particular, featured dystopian or apocalyptic scenarios in which the misuse of genetic science led to horrific mutations, biological warfare, and threatening clones, as well as oppressive surveillance states, which lessened autonomy and accentuated social and racial stratification. Literature and film also dramatized the significant impact that disclosures of genetic information can have on less powerful groups (women, LGBTQ+ individuals, and people of color), impacts that included diminishment of agency, stigmatization, discrimination, and eugenic measures aimed at people with genetic conditions. By contrast, TV shows in two genres—medical dramas and police procedurals—emphasized the value of genetics more than worries about privacy violations and dramatized the usefulness of medical genetics for diagnosis and treatment and of forensic genetics for law enforcement. Importantly, GetPreCiSe research was able to demonstrate the impact of popular culture on public attitudes toward genetic privacy by analyzing social media responses to works of literature, film, and television. Through studies of fan fiction, blog posts, Twitter posts, and reviews on Goodreads, researchers marshalled evidence that the positions that readers and viewers took on genetic privacy often stemmed from fictional works that they consumed.

As this account indicates, the GetPreCiSe project—again, made possible by the National Human Genome Research Institute’s innovative funding of Vanderbilt’s Center for Excellence in ELSI research—brought together a diverse group of scholars whose collaboration has provided numerous insights into the multifaceted implications of advances in genetics and genomics and the many related issues that require further exploration. These insights should be of benefit to policymakers, genetic scientists, doctors, lawyers and the general public in coming to conclusions about the collection, storage, and use of genomic information. This work also demonstrates the potential value of transdisciplinary work in thinking about the challenges posed by other new technologies, such as artificial intelligence and climate change.
